# Complement Factor H Expressed by Retinal Pigment Epithelium Cells Can Suppress Neovascularization of Human Umbilical Vein Endothelial Cells: An *in vitro* Study

**DOI:** 10.1371/journal.pone.0129945

**Published:** 2015-06-19

**Authors:** Yi Zhang, Qing Huang, Min Tang, Junjun Zhang, Wei Fan

**Affiliations:** Department of Ophthalmology, West China Hospital, Sichuan University, Chengdu, Sichuan Province, China; Sichuan University, CHINA

## Abstract

Complement factor H (CFH) is one of the most important soluble complement regulatory proteins and is closely associated with age-related macular degeneration (AMD), the leading cause of irreversible central vision loss in the elderly population in developed countries. Our study searches to investigate whether CFH expression is changed in oxidative damaged retinal pigment epithelium (RPE) cells and the role of CFH in the *in vitro* neovascularization. First, it was confirmed by immunofluorescence staining that CFH was expressed by ARPE-19 cells. CFH mRNA and protein in oxidative (H_2_O_2_) damaged ARPE-19 cells were both reduced, as determined by Real-time PCR and Western blotting analysis. Enzyme-linked immunosorbent assay (ELISA) also showed that ARPE-19 cells treated with H_2_O_2_ caused an increase in C3a content, which indicates complement activation. Then, wound assays were performed to show that CFH expression suppression promoted human umbilical vein endothelial cell (HUVECs) migration. Thereafter, ARPE-19 cells were transfected with CFH-specific siRNA and CFH knockdown was confirmed with the aid of Real-time PCR, immunofluorescence staining and Western blotting. The ELISA results showed that specific CFH knockdown in ARPE-19 cells activated the complement system. Finally, *in vitro* matrigel tube formation assay was performed to determine whether change of CFH expression in RPE would affect tube formation by HUVECs. More tubes were formed by HUVECs co-cultured with ARPE-19 cells transfected with CFH specific-siRNA when compared with controls. Our results suggested that RPE cells might be the local CFH source, and RPE cell injuries (such as oxidative stress) may cause CFH expression suppression, which in turn may lead to complement activation and promotion of tube formation by HUVECs. This finding is of importance in elucidating the role of complement in the pathogenesis of ocular neovascularization including choroidal neovascularization.

## Introduction

Increasing evidence shows that the complement system may play a significant but as yet undefined role in age-related macular degeneration (AMD), the leading cause of irreversible central vision loss in the elderly population in many industrialized countries. For this disease, choroidal neovascularization (CNV) is responsible for most of severe visual loss cases. One important AMD clinical hallmark at the early stage is drusen formation between the retinal pigment epithelium (RPE) and Bruch’s membrane. Some investigators have found that, on the basis of immunolocalization, there are many kinds of complement proteins and complement regulatory proteins (CRP) in drusen and CNV membranes [1–4]. This implies a potential relationship between complement systems and AMD, including CNV formation.

CRP can be divided into two types: soluble CRP and membrane linked CRP [5]. Complement factor H (CFH) is one of the most important soluble CRPs and an effective complement alternative pathway regulator. On human tissue surfaces, CFH combines with activated C3b and accelerates the C3 convertase inactivation process. CFH also works with complement factor I to inactivate complement systems [6]. In 2005 three independent research groups revealed the close relationship between CFH polymorphism and susceptibility to AMD [7–9]. Later, additional evidence showed that CFH dysregulation is closely associated with AMD. For example, during the course of CNV, CFH expression was down-regulated, and that caused CNV formation through up-regulating the expressions of vascular endothelial growth factor (VEGF), transforming growth factor beta (TGF-β), and MAC [10]. This result is consistent with a previous report that said complement components in drusen promote choroidal neovascularization [2].

Numerous studies with RPE cells, AMD animal models and humans have demonstrated that the RPE oxidative damage may be a trigger for the development of AMD [11]. RPE cells were found to secrete a variety of complement and complement regulatory proteins into drusen, including CFH [12]. It has been suggested that the formation of drusen involves complement activation and that the dysfunction of the RPE is an initiating event in complement activation [13]. These findings clearly support the overall hypothesis that RPE cells might be directly involved in local complement activation and AMD development. The potential relationship among RPE cells, complement and the formation of CNV needs to be further identified. In the present study, we investigated whether CFH expression is changed in oxidative damaged RPE cells, and how changes in CFH expression play a role in the *in vitro* neovascularization.

## Materials and Methods

### Blood collection and ethics statement

The study was approved by the Ethics Committee of the West China Hospital of Sichuan University and all aspects of the study comply with the Declaration of Helsinki. Blood samples were obtained from two healthy volunteers who are non-pregnant female adults and weigh at least 110 pounds. The total amount of blood drawn per volunteer is 20ml. Blood samples were drawn with single use needles into tubes without anticoagulant, which were purchased from Becton Dickinson. Because both blood donors were involved in the design of the project and performance of the experiment, and expressed understanding of the use of the serum, the procedures, and the potential risks and benefits, we consequently required only their verbal informed consent. This consent procedure was approved by the Ethics Committee of the West China Hospital of Sichuan University, considering that this procedure has no obvious harm to the participants. The basic information about the two volunteers, the informed consent process, the potential risks and the use of blood were all documented by another researcher and entered in a computer file. All of this information remains confidentiality and password protected.

### Cell culture

ARPE-19 (ATCC, Manassas, USA) cells were a generous gift from Prof. Kang Zhang’s lab (Shiley Eye Center, UC San Diego, California, USA). The cells were incubated in Dulbecco modified Eagle medium-Ham’s F12 (1:1) (Invitrogen, Carlsbad, California, USA) containing 10% fetal bovine serum (FBS) (Invitrogen, Carlsbad, California, USA) with 100 U/ml penicillin G and 100 mg/ml streptomycin at 37°C in a 5% humidified CO_2_ incubator to let the cells differentiate. ARPE-19 cells at passages 6–8 were used.

Human umbilical vein endothelial cells (HUVECs) were given as a gift by Prof. Kang Zhang’s lab (West China Hospital of Sichuan University, Chengdu, China) and incubated at 37°C in a 5% humidified CO_2_ with DMEM-high glucose containing 10% fetal bovine serum (FBS) (Invitrogen, Carlsbad, California, USA) with penicillin (100 U/ml) and streptomycin (100 mg/ml). The cells were confirmed to be vascular endothelial cells by their positive immunostaining for CD31. HUVECs at passages 4–6 were used.

### Human Serum Preparation

Blood samples were drawn into tubes without anticoagulant, followed by immediate refrigeration at 4°C refrigerator for 24 h to let the blood clot. Fresh human serum was then obtained from the blood samples and transferred into a clean tube. The serum was aliquoted to avoid freeze-thaw cycles and stored at -80°C.

### ARPE-19 cell exposure to oxidative stress

ARPE-19 cells were subcultured in 96-well plates, 48-well plates and 6-well plates at a density of 5×10^3^, 2×10^4^ and 3×10^5^ cells /well, respectively. After one day in culture, the cells reached about 80% confluence and were exposed to 400 μM H_2_O_2_ in culture medium without pyruvate for 24 h. This sub-lethal dose of H_2_O_2_ was chosen based on our preliminary experiments. The cells not treated with H_2_O_2_ served as controls.

### Cell viability assays

To assess the effects of H_2_O_2_ exposure on ARPE-19 cells viability, WST-1 assay (Roche, Basel, Switzerland) was performed according to the manufacturer’s instructions. After ARPE-19 cells were incubated with H_2_O_2_ for 24h as described above, 50μl WST-1 that was 10 times diluted was added and incubated for another 2 h at 37°C. Cells not treated with H_2_O_2_ served as controls. The optical density (OD) of the samples was measured at 440nm and 630nm respectively, using an enzyme-linked immunosorbent assay plate reader (Eppendorf, Hamburg, Germany). The cell viability was calculated based on the difference in OD between H_2_O_2_–treated and controls according to the equation shown below. The values are presented as percentages relative to the controls (defined as 100% cell viability). Experiments were carried out in triplicate and repeated three times independently. Cell viability (%) = (OD_440_—OD_630_ of treated cells/ OD_440_—OD_630_ of control cells) x 100%

### *In vitro* complement activation and C3a measurement

After ARPE-19 cells were treated with H_2_O_2_ as described above, the medium was aspirated. Subsequently, both the control and H_2_O_2_-treated RPE cells were washed twice with PBS and then incubated with 160μl human serum at 37°C for 2 h with soft agitation. To detect complement activation, enzyme immunoassay (Quidel Corporation, San Diego, California, USA) was used to measure the C3a content in the serum samples. 10μl serum was removed from each sample and diluted 5000 times. Serum samples from wells without RPE cells served as the background complement activation. The experiment was performed according to the manufacturer’s instructions and independently repeated three times, each analyzed in triplicate.

### RNA interference

ARPE-19 cells were grown in plates at a density of 150 cells/mm² and transfected with 50–150 nM CFH-specific siRNA (Santa Cruz Biotechnology Inc, California, USA), using a Lipofectamine2000 (Invitrogen, Carlsbad, California, USA) reagent, for 6 h according to the manufacturer’s instructions. Non-specific siRNA and a mock transfection control (without siRNA) served as controls. Cells were then cultured in basal media supplemented with FBS and growth factors for 24 or 48 h. CFH knockdown was determined with the aid of Real-time PCR, immunofluorescence staining, and Western blotting. For tube formation research, after being transfected with CFH-siRNA for 6 h, cells were cultured in basal media supplemented with FBS and growth factors for 48 h, and then cultured in media without FBS for 24 h.

### Quantitative real time reverses transcription-PCR

The ARPE-19 cells were harvested, and total RNA was isolated using Trizol reagent (Invitrogen, Carlsbad, California, USA). Reverse transcription was performed using 1 μg of total RNA, 0.25 μg of random primers (Sangon, China), and 1 μl of 10 mM dNTP mixture (Fermentas, Vilnius, Lithuania) according to M-MLV reverse transcriptase’s instructions (Invitrogen, Carlsbad, California, USA). For CFH, the forward primer was 5’-TACTGGCTGGATACCTGCTC-3’ and the reverse primer was 5’-CCTGACGGAGTCTCAAAATG-3’. The amplification conditions were: denaturation at 95°C for 5 s, annealing at 58°C for 5 s, and extension at 72°C for 30 s for a total of 40 cycles. Under optimized conditions there was a single melting curve and no primer-dimer formation. The housing keeping gene GAPDH was amplified to confirm there was no change in expression level under our experimental conditions, and it therefore served as an internal control. Experiments were performed in triplicate for each gene and were repeated three times using independent biological replicates. Gene Expression Analysis for iCycler iQ Real-Time PCR Detection System v1.10 (Bio-Rad, Hercules, California, USA) was used to analyze the gene expression level, and GraphPad Prism 4.0 was used to perform statistical analysis.

### Immunocytochemistry

ARPE-19 cells were plated on chamber slides or 24-well plates, and immunostaining for CFH was performed. After the indicated treatment above, RPE cells were washed twice with PBS, fixed for 10 minutes in 4% paraformaldehyde, permeabilized for 30 minutes in 0.1% Triton X-100, and washed three times with PBS. After blocking by 3% BSA for 1 h, RPE cells were incubated with the primary antibody (1:100 diluted in 1%BSA + 0.1% Triton X-100) overnight at 4°C. After being washed three times with PBS, cells were incubated with the secondary antibody (1:400 in 1%BSA + 1% Triton X-100) for 1.5 h in a dark environment at room temperature. After being washed five times with PBS, slides were mounted with antifade mounting medium with or without DAPI (Vector Laboratories, Burlingame, CA, USA) and were viewed with the aid of a fluorescence microscope at room temperature. Images were recorded with equal exposure conditions for each sample slide. The primary antibody was a goat antiserum raised against human Factor H (Quidel, San Diego, CA, USA), and the secondary antibody was donkey anti goat IgG antibody conjugated with Alexa Fluor 488 (Invitrogen, Carlsbad, California, USA). In some wells, primary antibody was omitted as a control for the specificity of secondary antibodies.

### Western blotting

Cultured ARPE-19 cell lysates were prepared using the RIPA lysis buffer (Tris HCl pH 7.4 50 mM, NaCl 150 mM, 1% NP40, 0.1%SDS, Na_3_VO_4_ 1 mM, NaF 1 mM) containing 1% proteinase inhibitor cocktail (Sigma-Aldrich, St. Louisst, Missouri, USA). Protein concentration was quantified using the BCA protein assay kit (Generay, Shanghai). Protein concentrations were adjusted to allow equal total protein loading on the gel. Fifty micrograms of protein from each sample were loaded and separated on the 8% SDS-PAGE gel. Proteins were then transferred electrophoretically to polyvinylidene difluoride membranes (Millipore, Bedford, Massachusetts, USA). Membranes were blocked for 4 h at 4°C in 0.1% Tween-20 Tris-buffered saline solution (TBST) containing 5% nonfat dry milk. They were then incubated with 1:200 goat polyclonal antibody (Quidel, San Diego, CA, USA) to human factor H in 2.5% non-fat milk and 2.5% BSA in TBST solution at 4°C overnight. After thorough washing with TBST, the membranes were incubated with 1:5000 HRP tagged mouse anti-goat IgG (Santa Cruz Biotechnology, Dallas, Texas, USA) at room temperature for 1 h and viewed by immobilon western chemiluminescent HRP substrate (Millipore, Bedford, Massachusetts, USA). β-actin was used as an internal control, and the bands’ gray scale was quantified by Quantity One software. The experiment was replicated 2–3 times using independent biological samples.

### Wound migration assay

ARPE-19 cells were exposed to oxidative stress in a 48-well as mentioned above. ARPE-19 cells without H_2_O_2_ treatment served as controls. Then, the medium was aspirated and 100μl human serum was added to each well, followed by incubation at 37°C for 2 h. HUVECs were plated at 2×10^4^cells /well in 48-well dishes at 37°C for 24 h. Wound assays were performed as previously described by Denker and Barber [14]. Briefly, wounds were made in HUVEC cultures using a pipette tip, and wounded monolayers were washed 3 times with PBS, followed by adding DMEM (without FBS, penicillin, and streptomycin) with the 20% human serum cultured with control or H_2_O_2_-treated RPE cells as mentioned above. Photographs were taken immediately (0 h) and 6 or 10 h after wounding. The distance the HUVEC monolayer migrated to close the wounded area was measured. Results were expressed as a migration index, the distance migrated by HUVECs at 6 or10 h relative to the width of the wound at 0 h. The experiment was carried out in triplicate and repeated three times.

### *In vitro* matrigel tube formation assay

For in vitro tube formation, matrigel (BD, Franklin Lakes, New Jersey, USA) was melted at 4°C and kept on ice. Then, 30μl matrigel was placed in each well of 96-well culture plates and immediately incubated at 37°C for 30 min to allow gel formation. After gel solidification, 3×10^4^ HUVECs were seeded on the polymerized gel in DMEM with 1% FBS. After incubation for 1.5 h at 37°C, the medium was aspirated, and the HUVEC gel was overlaid by an additional 30μl matrigel. After incubating at 37°C for 30 min, 5×10^4^ CFH-siRNA transfected or control ARPE-19 cells were placed over the HUVEC gel sandwich, and 100μl DMEM/F12 (without FBS, penicillin, and streptomycin) with or without 20μl human serum was added to each well. The co-culture was kept at 37°C for various time periods as indicated. Quantitative assays of tube formation under these conditions were performed at 24 and 72 h. Six different fields were chosen randomly in each well, and photographs were taken with a phase-contrast microscope. The tube lengths were measured using Image-Pro Plus 5.0 software (Media Cybernetics, L.P., Silver Spring, MD, USA) and expressed as an average length (μm) per microscopic field for each single well. For each independent experiment, at least three wells per condition were used, and each experiment was replicated three times.

### Statistical analysis

All data from quantitative assays were expressed as the mean ± standard deviation (SD). At least 2–3 independent repetitions with triplicate determinates were performed for each quantitative assay. Statistical analysis was performed using ANOVA or Student’s t-test with the aid of SPSS17.0. A significance cutoff of *P* < 0.05 was used.

## Results

### Cell cultures

**Fig 1:**
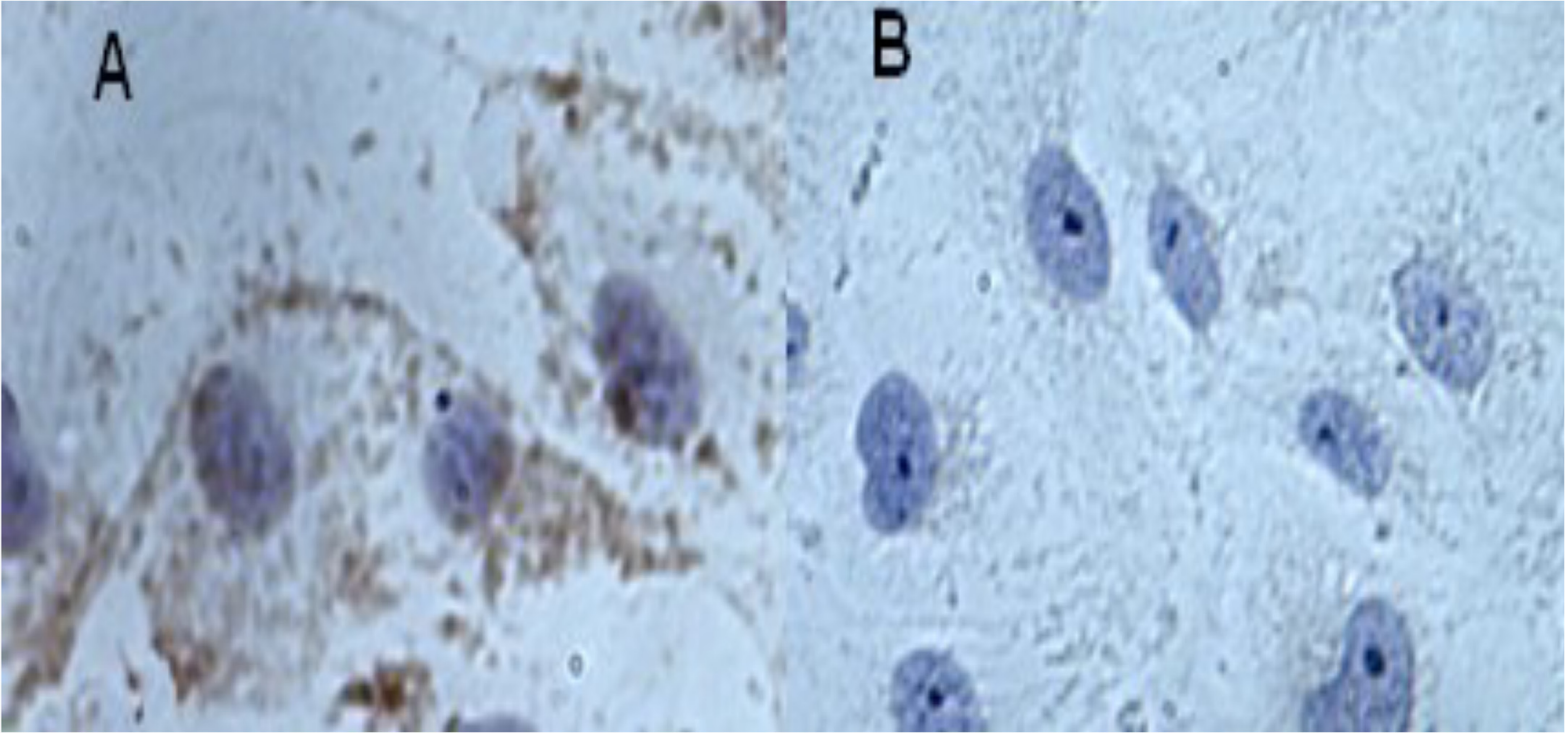
Cultured human umbilical vein endothelial cells (HUVECs) confirmed by immunohistochemistry. The cells have positive speckled immunostaining for CD31 (A) compare to Negative control (B).

HUVECs were confirmed to be vascular endothelial cells by their positive immunostaining for CD31 (Fig 1A). No immunostaining signal was detected in negative control (Fig 1B). Therefore, the cells were used in the following assays as source of vessel endothelial cells.

### CFH expression in ARPE-19 cells

**Fig 2:**
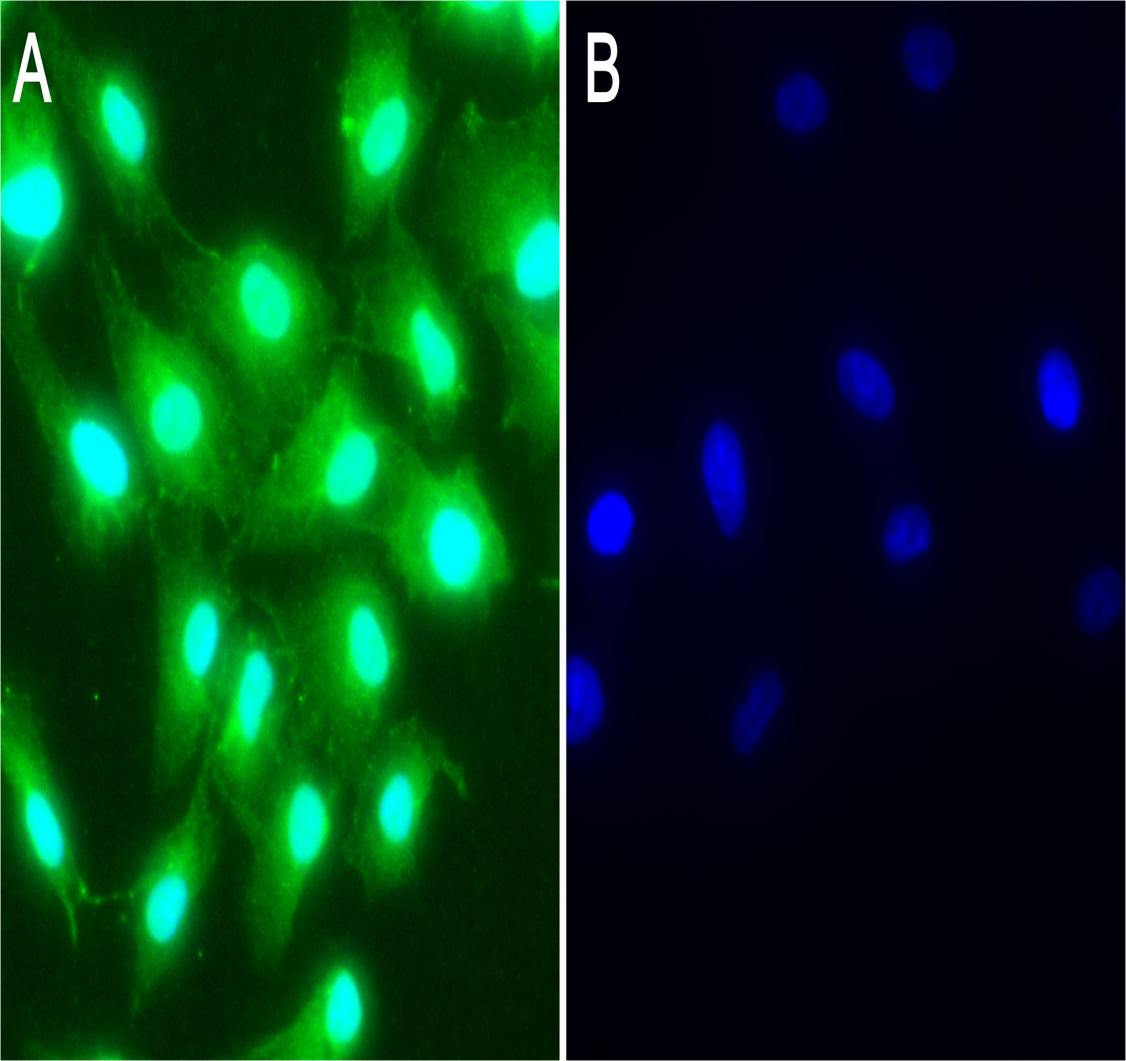
Expression of the CFH in ARPE-19 cells. The immunofluorescence results showed CFH expression (green) in the cytoplasm of ARPE-19 cells (A). Immunofluorescence staining was not detected in control wells where only secondary antibody was added (B).

CFH is expressed mainly in RPE in human and rodent eyes as well as in primary cultured RPE cells. To determine if CFH also exists in ARPE-19 cells, the cells were plated in 24-well plates or chamber slides and grew for 24 h (80% confluence) or longer to let the cell reach 100% confluence (up to 7 days). Immunofluorescence staining results showed that CFH was detected in ARPE-19 cells under confluent or sub-confluent (Fig 2A) conditions, indicating that the cell line may be used as a substitute for the primary cultured RPE cells for the following experiments. In negative control wells where the primary antibody was omitted and secondary antibody was directly added, no immunofluorescence signal was detected (Fig 2B), indicating the specificity of CFH staining.

### Effect of oxidative stress on ARPE-19 cell viability

**Fig 3:**
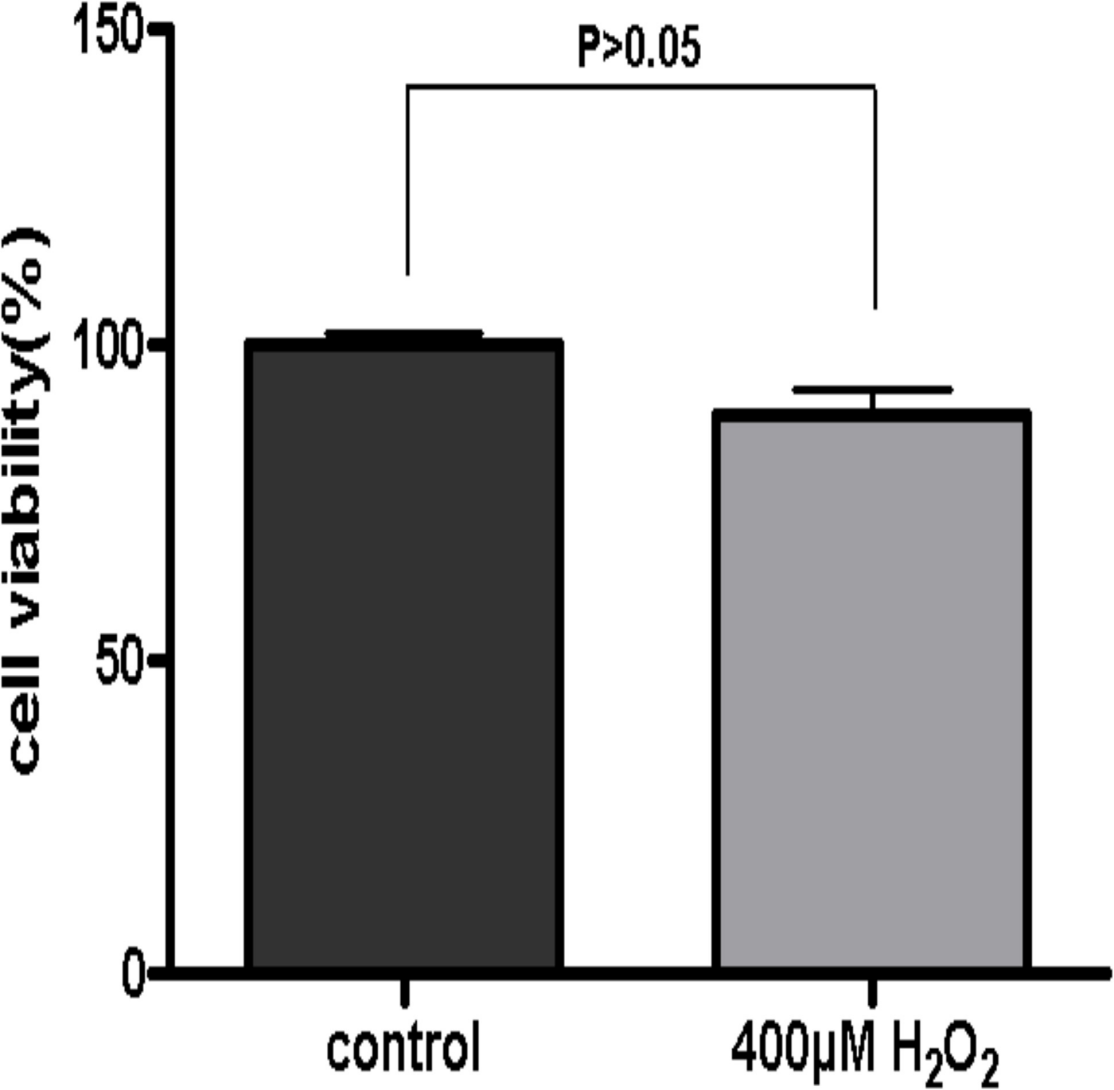
Cell viability of ARPE-19 cells. The viability of ARPE-19 cells was assessed by WST-1 assay. ARPE-19 cells were subjected to a single dose of 400μM H_2_O_2_ treatment for 24h and the untreated cells served as controls. The data showed that there was no significant difference in the cell viability between H_2_O_2_ treated and control cells (defined as 100%) (P > 0.05). The experiment was performed three times independently with triplicate in each group.

The cell viability of ARPE-19 cells subjected to 400μM H_2_O_2_ for 24h was tested with the aid of WST-1 assay. The results showed that there was no significant difference in cell viability between H_2_O_2_ treated and control groups (Fig 3, P > 0.05), although a minor decrease was detected in H_2_O_2_ treated ARPE-19 cells. Thus, this oxidative stress *in vitro* model was used in the following experiments.

### Oxidative stress in RPE cells reduced CFH expression

**Fig 4:**
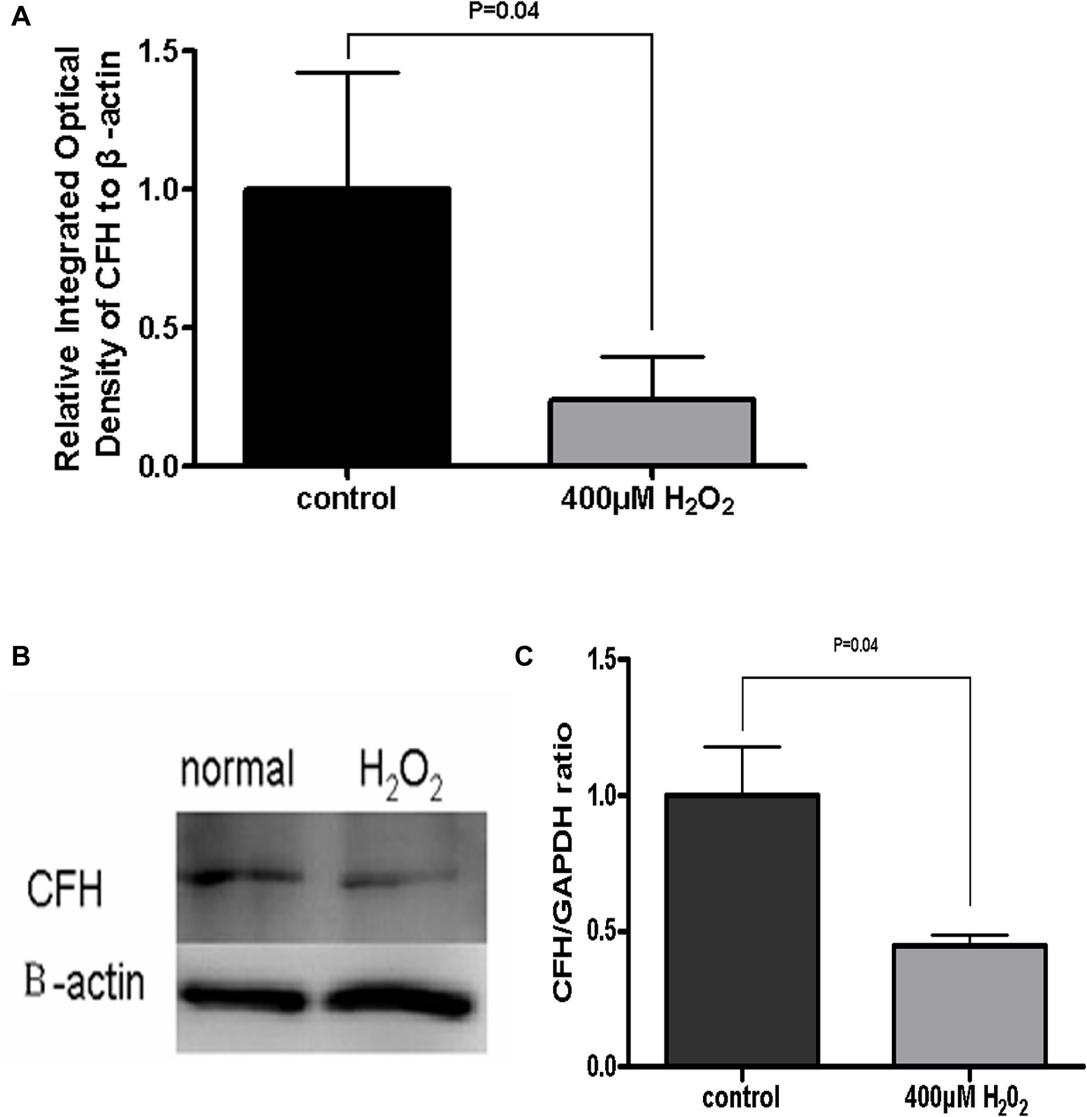
CFH expression reduced in the H_2_O_2_ treated ARPE-19 cells. Real-time PCR showed that the CFH mRNA expression in H_2_O_2-_damaged ARPE-19 cells was significantly lower than that in control cells (P < 0.05) **(A).** In Western blots, the CFH band of H_2_O_2-_damaged ARPE-19 cells was paler than the normal cells **(B).** The bands’ gray scale was quantified by Quantity One software. Compared to control cells, the CFH expression was reduced significantly in H_2_O_2-_damaged ARPE-19 cells (P < 0.05) **(C).** Data are presented as mean ± SD from results done with three independent biological samples, each analyzed with triplicates.

The main function of CFH is to down-regulate the alternative complement pathway. H_2_O_2_ has been demonstrated previously to reduce CFH expression in primary cultured RPEs [15]. In our study exposing ARPE-19 cells to 400 μM H_2_O_2_ for 24 h significantly reduced CFH mRNA by about 70% (Fig 4A). To further investigate whether the CFH protein was also affected by such oxidative stress in ARPE-19 cells, Western blotting was used to quantify the intracellular CFH proteins. CFH protein expression was reduced averagely by 55% after 24 h cell exposure to 400 μM H_2_O_2_ (P < 0.05) (Fig 4B and 4C). These data suggest that oxidative RPE cell injuries reduced CFH expression levels, which may play a role in triggering complement activation.

### Complement activation by oxidative stressed ARPE-19 cells

**Fig 5:**
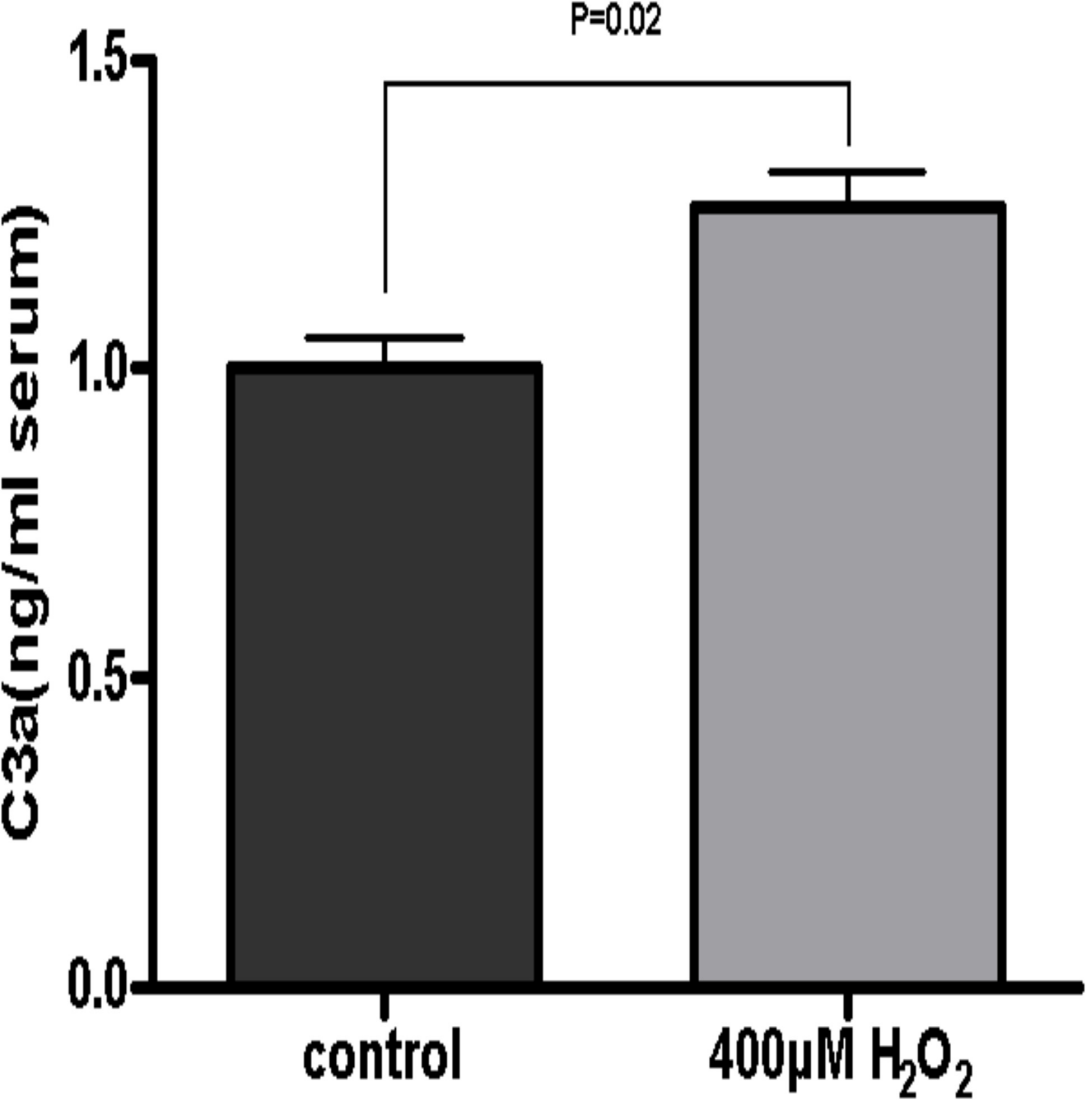
Complement activation *in vitro*. C3a content measured by ELISA in H_2_O_2_-damaged ARPE-19 cells was significantly higher than that in controls (P < 0.05). Results were expressed as mean ± SD. Experiments were carried out in triplicate for each condition and repeated three times independently.

To determine whether oxidative stress in ARPE-19 cells triggers complement activation, C3 cleavage was assessed *in vitro*. In these experiments, human serum was used as a source of complement [16]. We measured the C3a level in serum which contacted with ARPE-19 cells that had been treated with or without H_2_O_2_ to assess complement activation. C3a is generated by C3 cleavage and is then rapidly cleaved into the more stable C3a-desArg in serum [17]. Therefore the quantification of C3a-desArg provides a reliable method to measure the level of complement activation. Since a previous study reported that complement activation may occur when the overlying serum contacts the polystyrene well surface [17], we also assessed the complement activation background levels by measuring C3a after incubating serum in empty wells, and then ruled out this part in all other measurements. The ELISA data showed that treating ARPE-19 cells with H_2_O_2_ caused increased C3a content compared to controls (P < 0.05) (Fig 5), indicating that injured RPE cells may lead to complement activation.

### Effects of hydroperoxide-damaged ARPE-19 cells on HUVEC migration

**Fig 6:**
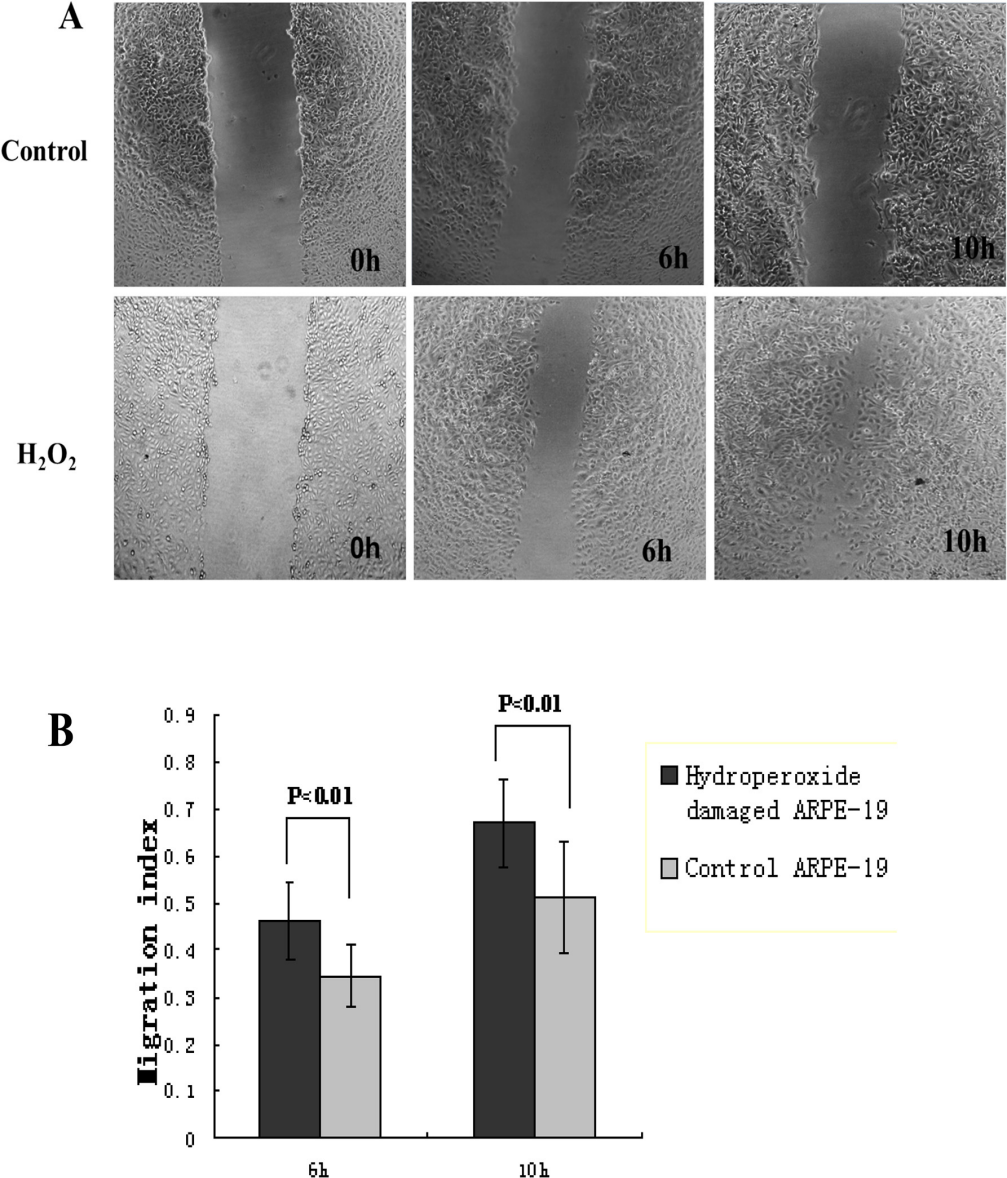
Effect of injured RPE cells on HUVEC migration. HUVEC migration was examined with a phase-contrast microscopy (×40) at the indicated time points (0h, 6h, 10h)(A). The wounding width was quantified, and bars represent the migration index of each treatment. The assay showed a significant increase of the distance moved by HUVECs treated with human serum that was cultured with H_2_O_2_-damaged ARPE-19 cells, when compared to HUVECs treated with the serum that was cultured with control ARPE-19 cells (P < 0.05) (B). The data were expressed as mean ± SD from results done with three independent biological samples, each analyzed with triplicates.

Our results showed that CFH protein expression in RPE cells was down-regulated by hydroperoxide damage and complement was activated by this damaged RPE cell, so we investigated whether human serum co-cultured with hydroperoxide-damaged ARPE-19 cells can mediate HUVEC migration. To rule out any effects of cell proliferation, we used DMEM without FBS and limited the observation time to less than 12 h. In wound-healing assays, HUVECs were treated with human serum that was cultured with damaged or control ARPE-19 cells. The distance moved by a wounded HUVEC cell monolayer on plastic was obtained with a phase-contrast microscopy (Fig 6A) and was quantified with Image-Pro Plus 5.0. Wound migration analyses showed a significant increase of the distance moved by HUVECs treated with human serum that was cultured with H_2_O_2_-damaged ARPE-19 cells, as compared to HUVECs treated with the serum that was cultured with control ARPE-19 cells (Fig 6B).

### CFH expression knockdown in ARPE-19 cells caused complement activation

**Fig 7:**
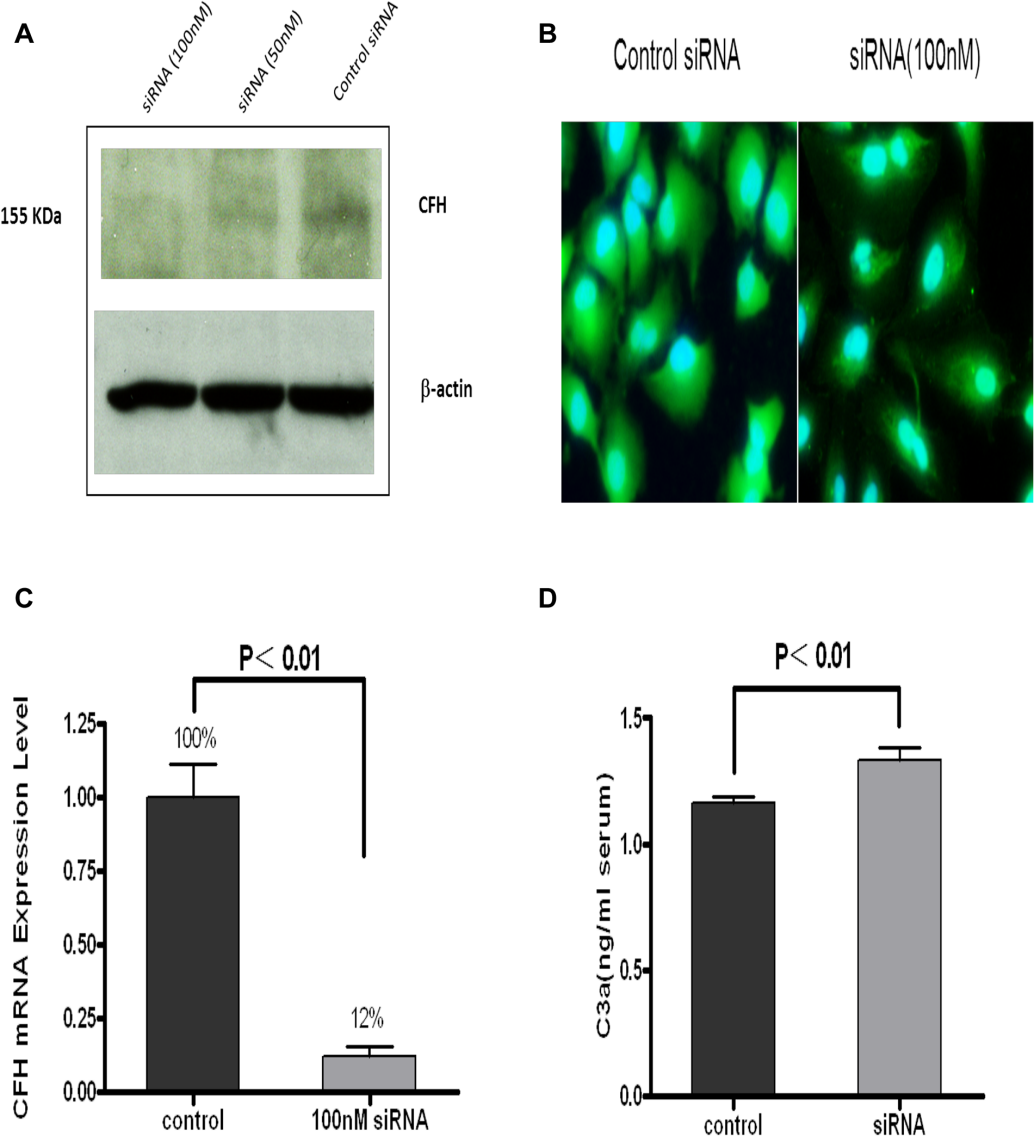
CFH RNA interference in ARPE-19 cells. (A) Western blot assay: CFH expression showed a reduction in RPE cells after being transfected with specific siRNA (100 and 50 nM siRNA) relative to the control (mock transfection with Lipofectamine reagent). The CFH expression level in the 100 nM CFH-siRNA transfected ARPE-19 cells was lower than that in the 50nM CFH-siRNA transfected cells. β-Actin served as an internal control. **(B)**. Immunofluorescence antibody labeling also showed that CFH immunostaining (green) in ARPE-19 cells transfected with 100 nM specific siRNA was weaker than in controls. The mock transfection served as controls. **(C).** Real-time PCR results showed CFH expression was knocked down by 88% using specific siRNA. Data are presented as mean ± SD (n = 9; T test; P < 0.01). **(D).** Complement activation: enzyme immunoassay confirmed that the C3a level in ARPE-19 cells after being transfected with specific siRNA (100 nM siRNA) was increased relative to the controls (mock transfection). Data are presented as mean ± SD (P < 0.01). All the experiments were carried out in triplicate for each condition and repeated three times.

To determine if reduced CFH expression in RPE causes complement activation, RNA interference was conducted to specifically knock down CFH expression in ARPE-19 cells. RPE cells were transfected with CFH-siRNA (50 nM and 100 nM) for 6 h. The specific CFH knockdown was evaluated at 24 to 48 h with Western blotting, Real-time PCR, and immunostaining. As shown in Fig 7A, compared with the controls, CFH expression was significantly inhibited by CFH-siRNA in a concentration-dependent manner. The inhibitory effect of the 100 nM siRNA was greater than the 50 nM siRNA. Fluorescence staining (Fig 7B) also showed a significant decrease in CFH expression in ARPE-19 cells treated with 100 nM CFH siRNA. This was further confirmed by Real-time PCR (Fig 7C), which showed that, compared to controls, CFH was knocked down by 88% with CFH-siRNA at a concentration of 100n M (T-test; P < 0.01). These data suggest that CFH-siRNA significantly reduced CHF expression levels in RPE cells.

Complement activation by RPE cells was assessed under specific CFH knockdown conditions. After RPE cells were transfected with CFH-siRNA at a concentration of 100 nM for 48 h, 160 μl of undiluted human serum was added to 48-well plates and incubated at 37°C for 2 h. Non-specific siRNA or monk-transfected RPE cells served as controls. As described above, C3a content was measured with ELISA in the incubated serum samples. As shown in Fig 7D, the C3a level in serum samples incubated with RPE cells with specific CFH knockdown was significantly higher than that in the controls (T test; P < 0.01). This finding indicated that specifically inhibiting CFH expression in RPE cells led to increased complement activation *in vitro*.

### Effects of CFH on tube formation in co-culture system

**Fig 8:**
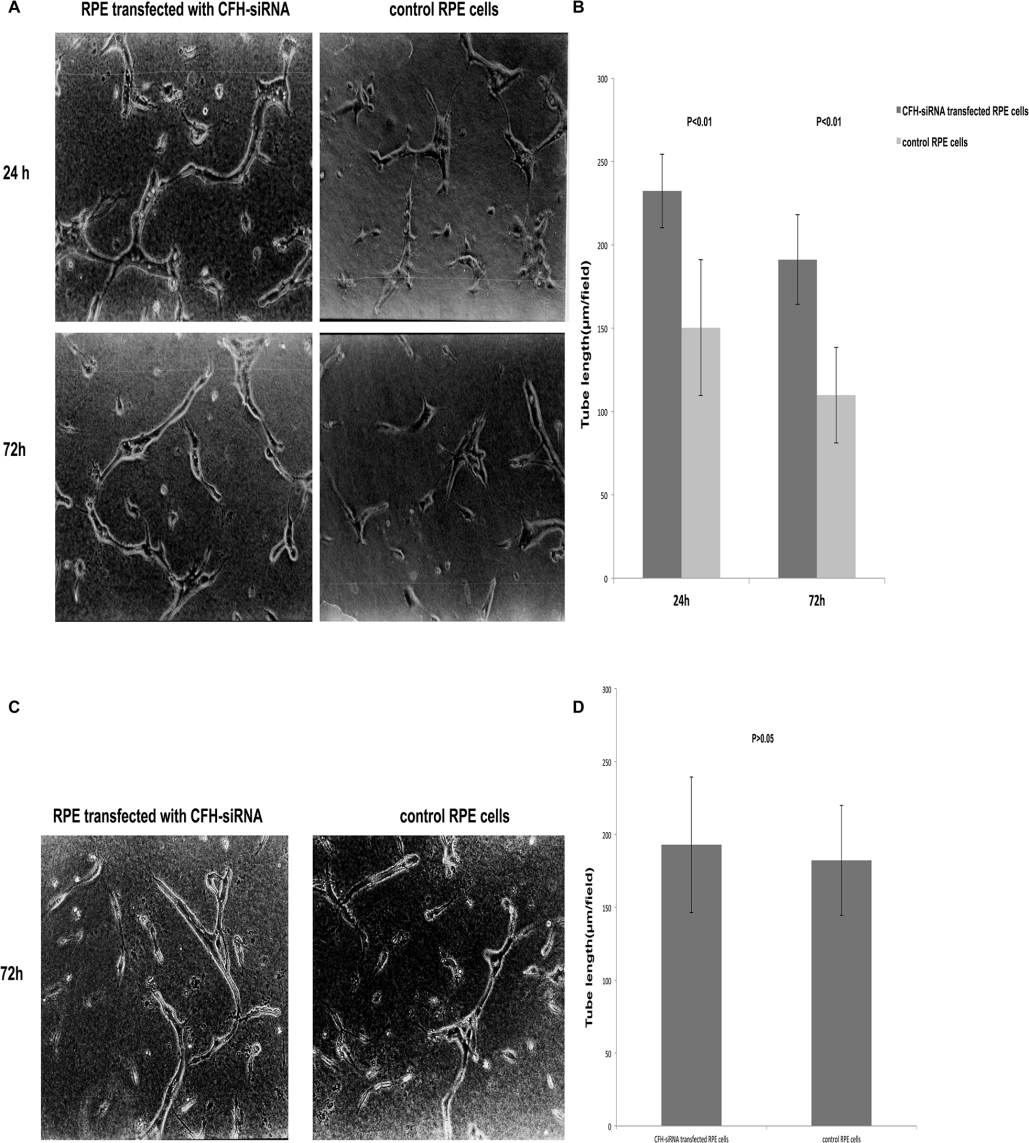
The tube formation by HUVECs. (A) and (B) showed that more tubes were formed by HUVECs in co-cultures with CFH-siRNA transfected ARPE-19 cells compared with control ARPE-19 cells at 24 and 72 h, respectively (P< 0.05). (C) and (D) showed that, in the absence of human serum, there were no significant changes in tube formation at 72h between CFH siRNA transfected RPE- and control non-transfected RPE-HUVEC co-cultures. Data were expressed as mean ± SD from results done with three independent biological samples, each analyzed with triplicates.

The ability of HUVECs to form vessels varied depending on the cell passages. More tubes formed when younger passage cells were used than when older passage cells were seeded in sandwich matrigel. In this study, HUVECs at passages 4–6 were used to ensure cells’ ability to form tubes both in control and experimental conditions. To test if changes of CFH expression level in RPE cells affect HUVEC tube formation, RPE cells with or without CFH knockdown with the RNA interference described above were seeded onto HUVEC sandwich gels in the presence or absence of human serum. The numbers of tubes were analyzed at indicated time points. As shown in Fig 8A and 8B, in the presence of serum more tubes were formed by HUVECs that were co-cultured with ARPE-19 cells transfected with CFH specific-siRNA, as compared to those in the control co-cultures where control RPE cells were used. However, in the absence of human serum, there were no significant changes in tube formation between CFH siRNA transfected RPE- and control non transfected RPE—HUVEC co-cultures (Fig 8C and 8D). These findings suggest that CFH may not directly affect tube formation in the assay system; instead, it may play a role through complement activation.

## Discussion

AMD is the leading cause of irreversible blindness in the elderly, and the most devastating disease complication is CNV, the hallmark of wet AMD. Several pathogenesis theories have been proposed for this complex and multifactorial aging disease. Oxidative stress is implicated in AMD’s pathophysiology, and the presence of such stress has been shown through lipid peroxidation markers and antioxidant enzyme activities [18]. Oxidative stress causes injury to the RPE and several studies have shown that RPE cell damage is closely related to the CNV formation. An and colleagues [12] found that, compared to normal RPE, RPE in AMD patients not only secreted more complement regulatory protein, but also secreted more protein related to tissue development and new vessels. This indicates that RPE may be involved in regulating local complement activation and neovascularization. Zhou [17] reported that A2E oxidation products may induce RPE oxidative damage and can activate the complement system. These findings are consistent with our results. Our data showed that treating ARPE-19 cells with H_2_O_2_ caused increased C3a content compared to controls, indicating that oxidative stressed RPE cells may lead to complement activation.

In this study exposure of ARPE-19 cell to 400μM H_2_O_2_ for 24 hours in the presence of serum in medium caused no significant change in cell viability. This was an important premise for all of the other experiments relating to oxidative stress on the RPE cell and its function assays. Our data are supported by previous studies, in which cell viability of ARPE-19 cells was not affected under similar experimental conditions[19]. However, controversial data were also reported in some other studies [20, 21], which showed an approximate 40–50% decrease in ARPE-19 cell viability after H_2_O_2_ treatment at similar concentrations. We considered this inconsistency to be mainly due to the difference in experimental conditions used in those studies, including serum-free medium, cell density and passages, as well as methods of assays, etc. Another important issue that needs to be addressed is that the H_2_O_2_ treatment used in this *in vitro* assay was an acute oxidative injury. It is not completely similar to the condition when chronic oxidative damage occurs in *in vivo* AMD pathology, where chronic oxidative stress finally cause cell apoptosis [22]. Since H_2_O_2_ generates significant intracellular reactive oxygen intermediates (ROIs) that are degraded enzymatically, and are thus short-lived in the cell [23], a chronic oxidative stress model of RPE cells needs to be developed, in order to better simulate the *in vivo* situation in future research.

Recent research researches showed that complement activation and inflammation is important in AMD mechanisms [4, 24–26]. Various kinds of complement components, inflammatory associated molecules and complement regulatory proteins are deposited in drusen [3, 5, 27–29]. Complement is a major component of innate immunity, and a number of complement regulators, either soluble or membrane-bound, have been implicated in preventing full-blown immune system activation [1]. The basic structure of all three complement pathways (alternative, lectin, and classical) is initiated by different mechanisms, and then C3 convertase forms and cleaves C3 to C3a and C3b. Complement C3b functions cooperatively to form C5 convertase, which cleaves C5, and finally forms the membrane attack complex (MAC) [30]. CFH is one of the most important soluble CRPs and an inhibitor of complement activation in the alternative pathway. CFH can regulate the C3 convertase by promoting degradation of complement components C3b to protect the host cell against autologous attack and reduce C3 and C5 convertase formation. In human and rodent eyes, CFH is locally and primarily expressed in RPE [31]. The CFH gene mutation may impair its inhibitory function and cause complement alternative pathway activation and local inflammation, which may lead to AMD development [6, 32, 33]. CFH is not only an essential part of AMD pathogenesis but also affects CNV treatment. The CFH Y402H gene variant was shown to be associated with photodynamic treatment (PDT) for CNV and seemed to affect visual outcomes after PDT treatment. The CFH CC genotype increases PDT risk and the degree of vision loss following PDT [34]. Other researchers have also proved that patients with the CFH CC genotype responded significantly worse after intravitreal bevacizumab than did those with the TC and TT genotypes [35]. Thus, CFH may be an important molecule for studying AMD pathogenesis and a target for developing further treatment strategies.

The RPE is the major site for CFH expression in the eye. RPE is exposed to various injuries *in vivo*, including oxidative stress, which has been shown to be related to AMD. RPE oxidative damage may down-regulate CFH expression and impair its inhibitory role in complement system activation [15, 36]. Our research investigated the complement regulatory protein CFH expression level in ARPE-19 cells after being exposed to oxidative stress. This study showed that exposing ARPE-19 cells to H_2_O_2_ reduced both CFH mRNA and CFH protein expression levels and activated complement activation as well. These findings further demonstrated that RPE damage may be a trigger for complement activation and down-regulation of CFH, a major inhibitor of alternative pathway, which may be involved in this mechanism.

To reveal the possible role of RPE and its CFH expression in neovascularization pathogenesis, wound migration assay was used. Our study suggested that RPE damage and CFH suppression by hydroperoxide stress promoted HUVEC migration probably through complement activation. Since endothelial cell migration is an essential step in forming neovessels, our findings point to a possible role of injured RPE, its decreased CFH expression level and increased complement activation in neovascularization pathogenesis. Because previous studies have showed that H_2_O_2_ treatment also increased VEGF in RPE *in vitro* [37], the role of such factors on complement activation and HUVEC migration cannot be excluded in this assay. Therefore, we also sought to investigate the specific role of CFH expression in RPE in complement activation and neovascularization.

Employing the aid of RNA interference, this research study has proven that CFH expression was significantly inhibited by CFH-siRNA in a concentration-dependent manner, suggesting a specific suppression of CFH in RPE cells by siRNAs. Furthermore, CFH knockdown in RPE by RNA interference can also activate the complement system. To our knowledge, this is the first study to demonstrate the direct role of CFH expression level in RPE in regulation of complement activation. This is of importance when taking into account of the following facts and findings that: 1) CFH is an inhibitor of complement activation and is involved in AMD pathogenesis; 2) CFH is mainly expressed in RPE in the eye; 3) RPE is close to choroidal vessels; and 4) any damage to RPE cells may cause local complement activation which may be a trigger for local inflammation and result in CNV formation. Indeed, Lundh von and colleagues found injecting short interfering RNA (siRNA) which targets CFH beneath the retinal space may inhibit local CFH expression and increase local MAC deposition. This will deteriorate laser-induced choroidal neovasculation, which may reveal a role of CFH underlying the pathological mechanism of wet AMD [33]. So, taking together these findings, we may conclude that the expression of CHF on RPE may have an important role in regulating local complement activation and CNV formation.

To further test if specific knockdown of CFH in RPE cells and complement activation play a role in neovascularization, we used an *in vitro* tube formation system in which RPE cells and HUVEC were co-cultured in matrigel. The advantage of this co-culture model is that a stable RPE monolayer has been placed over a matrigel into which HUVECs have been seeded and thus can provide a more natural assay for studying RPE–EC interactions [38]. In this RPE-HUVEC co-culture model, we demonstrated that specific CFH knockdown in RPE induced more tube formation in the presence of human serum. This seemed to be related to more complement activation resulting from CFH down-regulation, since there was no significant difference in tube formation when human serum was absent in the co-culture system. The underlying mechanisms of CFH knockdown and complement activation in neovascularization are not completely known yet. Some researchers showed that the C5a receptor is expressed in the human choroid and retina *in vivo* and will cause an increase in the ratio of the S/G2/M cell-cycle phases in cultured vascular endothelial cells. C5a can even increase microvascular-like structure formation in matrigel plug assay [39, 40]. Other researchers proved when aortic endothelial cells (AECs) were treated with MAC, a significantly large number of cells progressed from the G0/G1-phase to the S-phase and G2/M-phase. However, the more important feature MAC has is that it can induce various kinds of endothelial cells to release growth factors like VEGF, TGF-β2, and β-FGF *in vitro* and *in vivo* experiments [3, 41, 42]. Based on these results, we assumed the mechanism CFH uses to suppress neovascularization might be affected by decreasing C3a, C5a, and MAC formation so as to decrease growth factor secretion, but this hypothesis needs to be further studied in future research. Whether CFH has a direct function on neovascularization pathogenesis also warrants additional study.

Since whole human serum was used as a source of complement in the co-culture assays, MAC formation and cell attack should be considered. Previous studies have shown that several factors inhibiting MAC formation are expressed at high levels on these two kinds of cells, such as CD59 on HUVECs[43] and CD 55, CD59 and CD46 on RPE and human choroidal endothelial cells[44–46]. These molecules may protect the cells against MAC mediated cytotoxicity, so we speculate that few cells are attacked by MAC and that the issue will not affect our research results. As expected, we did not find any significant cytotoxicity of human serum on either RPE or HUVEC cells in our co-culture system.

Although we have proven that RPE damage causes decreased CFH expression which may lead to complement activation and tube formation by HUVEC *in vitro*, implying a role of damaged RPE as a trigger to activate local complement and to induce CNV formation, cautions should be taken when making conclusions about *in vivo* conditions, since more complicated molecular mechanisms and pathways are involved, such as VEGF, TGF-β, insulin-like growth factor (IGF)-I, and pigment epithelium derived factor (PEDF) etc. [47–50], just to mention a few. All of these factors contribute to the development of choroidal neovascularization and show their function by interacting with each other. Complement activation also has interactions with these angiogenic/antiangiogenic factors and other pathological processes including inflammation and others.

In summary, we found that the expression level of CFH, an inhibitory of complement activation, was reduced in oxidative-damaged RPE. This effect increased the complement activation and may be one of the pathological mechanisms underlying AMD development. We also were able to prove that CFH expressed by RPE cells suppresses tube formation, a neovascularization-like process in a co-culture model. More studies are needed to determine how CFH and complement functions in neovascularization, specifically in CNV, and hopefully can provide with new insight into the pathogenesis of this disease and develop more treatments for this disease.
